# Polygenic risk score for embryo selection—not ready for prime time

**DOI:** 10.1093/humrep/deac159

**Published:** 2022-07-19

**Authors:** Alex Polyakov, David J Amor, Julian Savulescu, Christopher Gyngell, Ektoras X Georgiou, Vanessa Ross, Yossi Mizrachi, Genia Rozen

**Affiliations:** Faculty of Medicine, Dentistry and Health Sciences, University of Melbourne, Melbourne, VIC, Australia; Reproductive Biology Unit, The Royal Women’s Hospital, Parkville, VIC, Australia; Melbourne IVF, East Melbourne, VIC, Australia; Murdoch Children’s Research Institute, Parkville, VIC, Australia; Department of Paediatrics, Royal Children’s Hospital, University of Melbourne, Parkville, VIC, Australia; Oxford Uehiro Centre for Practical Ethics, Faculty of Philosophy, University of Oxford, Oxford, UK; Biomedical Ethics Research Group, Murdoch Children's Research Institute, Melbourne, VIC, Australia; Melbourne Law School, University of Melbourne, Melbourne, VIC, Australia; Melbourne Law School, University of Melbourne, Melbourne, VIC, Australia; Department of Paediatrics, University of Melbourne, Melbourne, VIC, Australia; Reproductive Biology Unit, The Royal Women’s Hospital, Parkville, VIC, Australia; Faculty of Medicine, Dentistry and Health Sciences, University of Melbourne, Melbourne, VIC, Australia; Reproductive Biology Unit, The Royal Women’s Hospital, Parkville, VIC, Australia; Melbourne IVF, East Melbourne, VIC, Australia; Reproductive Biology Unit, The Royal Women’s Hospital, Parkville, VIC, Australia; Faculty of Medicine, Dentistry and Health Sciences, University of Melbourne, Melbourne, VIC, Australia; Reproductive Biology Unit, The Royal Women’s Hospital, Parkville, VIC, Australia; Melbourne IVF, East Melbourne, VIC, Australia

**Keywords:** polygenic risk score, embryo selection, preimplantation genetic screening, ethics, assisted reproduction

## Abstract

Numerous chronic diseases have a substantial hereditary component. Recent advances in human genetics have allowed the extent of this to be quantified via genome-wide association studies, producing polygenic risk scores (PRS), which can then be applied to individuals to estimate their risk of developing a disease in question. This technology has recently been applied to embryo selection in the setting of IVF and preimplantation genetic testing, with limited data to support its utility. Furthermore, there are concerns that the inherent limitations of PRS makes it ill-suited for use as a screening test in this setting. There are also serious ethical and moral questions associated with this technology that are yet to be addressed. We conclude that further research and ethical reflection are required before embryo selection based on PRS is offered to patients outside of the research setting.

## Introduction

The birth of Louise Brown in 1978 (Steptoe and Edwards, 1978) following IVF ushered in a new era in human reproduction and had a profound impact on society in general. In countries that use this technology liberally up to 5% of children are born through ART (Chambers *et al.*, 2017). Advances in the understanding of human genetics allowed for embryo selection to avoid various genetic conditions. Outside of embryo selection, wide availability of affordable genetic sequencing allowed the proliferation of private enterprises that offer this service for non-medical indications, such as genetic genealogy (Kling *et al.*, 2021). Availability of large population-based genetic databases has enabled diseases with only a partial genetic basis to be evaluated in terms of future risk and individuals to be risk stratified based on their unique genetic profile. This has given rise to what is termed a ‘polygenic risk score’ (PRS) where a particular combination of genetic variants may confer a specific likelihood of developing a certain polygenic condition, such as hypertension (Tada *et al.*, 2021) or diabetes, later in life (Konuma and Okada, 2021). Furthermore, PRS can be derived for various non-disease-related parameters, including intelligence, height, endurance and even criminality (Kendler *et al.*, 2019). This technology has recently been applied to embryo selection (Treff *et al.*, 2019a), which raises significant ethical concerns (Lázaro-Muñoz *et al.*, 2021). This article discuss the concept of PRS, its proposed use in embryo selection as a screening test and ethical issues related to such use.

## Background to PRS

PRS calculations rely on a complex statistical modelling of disease risk, based on genome-wide association studies (GWAS), using single-nucleotide polymorphisms (SNPs). SNPs are variations in nucleotide sequence that differ between individuals, and each SNP may be associated with a small change in the risk of one or more common diseases (Manolio *et al.*, 2009). Since an individual SNP only confers a small variation in the disease risk, a combination of SNPs is required to produce a model which meaningfully assesses an individual’s risk. This is the theoretical basis of PRS, where multiple SNP-associated risks are combined into a polygenic model (Golan *et al.*, 2014) that can then be applied to a particular individual.

It is important to consider how individual SNP risk of disease is generated. At the most basic level, a GWAS dataset, that comprises the clinical characteristics and SNP profiles of a study population, is obtained. In essence, the study population is divided into those who develop and do not develop the disease being studied. The difference in SNPs is then examined to determine which SNPs increase, or decrease, the risk of the disease (Lewis and Vassos, 2020).

## Preimplantation genetic testing

Preimplantation genetic testing (PGT) involves obtaining a small sample from the trophectoderm of a developing embryo. Once a DNA sample is extracted from the biopsy, it is amplified for genetic analysis. Multiple investigations can be undertaken, potentially including characterization of the embryo’s SNPs profile (Treff *et al.*, 2019b). Some applications of PGT are well established, while others are controversial in terms of effectiveness, especially in the setting of screening for numerical chromosomal aberrations (Munné *et al.*, 2019; Pagliardini *et al.*, 2020), as well as to exclude embryos that may produce non-lethal phenotypes, such as deafness or dwarfism, regarded by some disability advocates as non-pathological variants (Wallis, 2020).

It was only a matter of time before PRS analysis was applied to embryo selection. A number of companies based in the USA, such as Orchid Biosciences and Genomic Prediction, offer PRS for embryo selection (PRS-ES) analysis to prospective parents (Genomic Prediction Clinical Laboratory, 2022; Orchid Health, 2022). In a minimally regulated U.S. ART market, these companies promote their services directly to consumers and promise “advanced embryo screening” for such diverse conditions as Alzheimer’s disease, stroke, breast cancer and diabetes, among others. There are significant concerns, which render such claims suspect and the introduction of this technology for embryo selection, ethically questionable. These concerns mainly relate to the overall value of such testing in terms of its ability to deliver promised outcomes, as well as the social and ethical implications of widespread adoption of this novel technology should it prove to be beneficial to individual patients.

## PRS as a screening test for embryo selection

PRS-ES is a screening test and should be evaluated as such before its widespread use can be advocated. There are well established frameworks to evaluate the acceptability, ethical and otherwise, of population-based screening tests. The most widely used criteria to evaluate screening tests were developed in the 1960s by Wilson and Jungner (1968). With some modifications, it has been applied to a variety of screening scenarios, including newborn screening and genetic screening (Petros, 2012; King *et al.*, 2021). A further refinement by Botkin produced an ACCE acronym which refers to A: analytic validity, C: clinical validity, C: clinical utility, E: ethical, legal and social implications (Botkin, 2009). This framework can, and should, be applied to evaluate the utility of PRS-ES. The basic questions that need to be answered before introducing any test or novel technology is whether it will achieve the outcomes promised and whether the benefits outweigh the risks (Table I).

**Table I:** Polygenic risk score for embryo selection: an evaluation summary.

Screening test evaluation components (ACCE^*^)	PRS-ES^**^ issues identified	PRS-ES overall assessment
Analytic validity	Partially established (Treff *et al.*, 2019b), but unresolved issues remain (Zong *et al.*, 2012; Peters *et al.*, 2015; Delaneau *et al.*, 2019).	May be acceptable, but further research required to address remaining concerns.
Clinical validity	Cannot be demonstrated owing to temporal disconnect between the test and outcomes. Changes in environmental conditions likely to make PRS-ES clinically invalid.	PRS-ES is unlikely to be clinically valid.
Clinical utility	Overall improvement in clinical outcomes is impossible to demonstrate. Unlikely to be clinically significant owing to the limited number of embryos available for analysis and significant uncertainties inherent in PRS-ES. Clinical utility may be further decreased if the gender of an embryo is taken into account.	Clinical utility of PRS-ES is highly questionable, cannot be demonstrated by acceptable research protocols.
Ethical, legal and social implications	Multiple ethical issues and social implications remain unresolved. Possible harms and questionable benefits, especially if IVF and embryo biopsy are undertaken for the sole purpose of PRS-ES.	Cannot be considered ethically justified.

* ACCE—analytic validity, clinical validity, clinical utility, ethical implications.

** PRS-ES—polygenic risk score for embryo selection.

## Analytic validity

This refers to the accuracy with which a genetic characteristic, such as SNPs, is identified by a given test (Burke *et al.*, 2002). There is little doubt that currently employed techniques of genetic analysis are accurate and reliable. The SNPs analysis is usually a true reflection of the genetic makeup of the cells being analysed, barring any laboratory mishaps. There is also at least one study that demonstrates the possibility of PRS estimation, concurrently with the more established genetic evaluations of an embryo (Treff *et al.*, 2019b). There are, however, well recognized technical limitations when comprehensive profiling of an embryo’s genome is attempted. These are related to the limited quality and quantity of DNA available from a small number of cells derived from a trophectoderm biopsy (Zong *et al.*, 2012; Peters *et al.*, 2015). Furthermore, statistical manipulation of genetic data may limit the detection of rare pathogenic gene variants (Delaneau *et al.*, 2019). Novel approaches are being developed to address these limitations and some report excellent concordance between embryo biopsy and post-natal genetic analysis (Kumar *et al.*, 2022). These remain expensive, labor-intensive and what can only be classified as being ‘in development’ stage of their evolution, with no published experience of wide implementation. Common genetic variations found in the trophectoderm of human embryos have not been systematically addressed and may create an additional level of uncertainty, as is acknowledged by the authors of the latest study (Kumar *et al.*, 2022). It must be concluded that the analytic validity of PRS-ES is currently questionable, and it does not fulfil this criterion for widespread implementation, outside of the research setting. Further investigative efforts in this area should be directed towards demonstrating concordance of PRS-ES with post-natal genetic evaluations.

## Clinical validity

This criterion was proposed in 1999 to describe the ability of a genetic test to identify a clinical condition in question (Holtzman and Watson, 1998). The overall accuracy of a test is commonly assessed by traditional epidemiological parameters, including positive and negative predictive values, as well as sensitivity and specificity. In the context of PRS-ES, it is a measure of how well a genetic variant being analyzed is related to the risk of a specific condition developing in the distant future. This question cannot currently be answered because of the temporal disconnect between the proposed testing and the outcomes of interest. However, there are strong reasons to suspect that PRS-ES may lack clinical validity. This is related to the methods used to calculate PRS (Wald and Old, 2019), which may make them ill-suited to be used for embryo selection. One must remember that complex multifactorial conditions, such as the ones where PRS are used, have a substantial environmental component. Risk factors, such as obesity, smoking, diet, environmental exposure to toxins and general living conditions, play an important role in disease pathogenesis. There is no doubt that a disease phenotype is the outcome of a lifelong interaction between one’s genes and the environment. It is important to note that PRS are derived from studies of people who have lived long lives in certain environments. It is also undoubtedly true that humans conceived today will live their lives in a radically different environment compared to people who were born 50 years ago. In the past 100 years, environmental conditions and lifestyle have changed dramatically, and these changes have accelerated in recent decades. The gene–environment interactions that produced a disease phenotype in people in their 50s and 60s are unlikely to be similar to the gene–environment interplay over the next 50 years. Thus, not only is it difficult to assess the clinical validity of PRS-ES in terms of the outcomes in question, it is also possible that clinical validity would be limited by the different effects of future environment on gene expression, compared to the past. This represents one of the inherent limitations of PRS, where the original study population may have been under different environmental influences compared to the populations to which PRS is applied (Khoury *et al.*, 2013).

## Clinical utility

In the context of screening, the clinical utility measure may most accurately reflect the overall value of a genetic test. It is defined as the evidence that a genetic test improves clinical outcomes in a measurable way. A test must be useful in terms of aiding clinical decision-making compared to a currently utilized strategy without screening (Teutsch *et al.*, 2009). On the most fundamental level, clinical utility refers to the likelihood that the test will lead to an improved health outcome (Burke *et al.*, 2002). Considering the inherent limitations of PRS even for their original purpose, namely risk stratification on an individual and population level of people who are currently at risk of developing the diseases in question (Wald and Old, 2019), it appears highly unlikely that PRS-ES of people yet to be born will be of substantial clinical utility. Furthermore, it is difficult to meaningfully assess the clinical utility of a test for a condition which may or may not manifest itself decades into the future. Mathematical modelling of PRS-ES has been attempted and indicated extremely limited utility in terms of non-pathological trait selection (Karavani *et al.*, 2019), such as height and intelligence quotient (IQ). This is also true for disease traits such as Crohn’s disease and schizophrenia (Lencz *et al.*, 2021).

It appears that prospective parents are commonly being misled by the companies advertising this service by quoting relative risk reduction rates, without putting these values into appropriate perspective (Turley *et al.*, 2021). As elegantly described by Turley *et al.* (2021), the purported benefit of PRS-ES is commonly calculated and presented as a difference not between two average embryos, but rather a difference between the highest and the lowest possible risk embryos, thus maximizing the theoretical benefit of the test. The value of PRS-ES may also depend on the selection strategy applied (Lencz *et al.*, 2021), but these nuances are not clearly communicated to the prospective patients. There are numerous other reasons as to why the clinical utility of PRS-ES may be of limited value. They relate to the assumptions inherent in PRS modelling, which may not be applicable to the population assessed. There is also the reality of the IVF process, which usually produces only a limited number of embryos that are suitable for genetic screening. This makes the choice of embryos available rather limited in most instances. It is also important to note that all embryos produced by a couple are genetically related and share on average 50% of SNPs. One must conclude that owing to inherent limitations of the PRS-ES models and limited variation in the genetic makeup of embryos produced by a couple, the clinical utility of PRS-ES is almost certainly diminutively small (Karavani *et al.*, 2019).

Another surprisingly unexplored issue relates to the impact of an embryo’s gender on PRS. There are conditions that affect only one gender, such as cervical, uterine, ovarian and prostate cancer. There are conditions that are much more prevalent in one sex, such as breast cancer. Most of the conditions which can be assessed using PRS have a significant gender association. This implies that by selecting one sex over the other, a prospective parent can influence the risk of all the conditions in question. Furthermore, sex-based risk is likely to be more significant compared to PRS-ES calculated risk. This can be demonstrated by looking at the list of conditions listed on the website of a PRS-ES provider (Orchid Health, 2022) as being part of the PRS calculation. The following conditions are significantly more prevalent in males: schizophrenia, heart disease, atrial fibrillation, stroke, prostate cancer and type 2 diabetes. The only conditions with higher prevalence in females are breast cancer and Alzheimer’s disease, while inflammatory bowel disease and type 1 diabetes do not appear to be strongly influenced by gender (Mokdad *et al.*, 2018). Therefore, by choosing a female embryo, one can substantially reduce the overall risk of developing most diseases included in this list. Conversely, by choosing an embryo with a lower PRS, one is more likely to select a female embryo and, in fact, the first baby born utilizing PRS-ES was a female (Bloomberg News, 2021). It is also important to note that owing to a variety of factors that are beyond the scope of this paper, males appear to have higher overall quality of life (Orfila *et al.*, 2006), while at the same time having lower life expectancy, compared to females (Australian Institute of Health and Welfare, 2021). Poor diet, limited exercise and tobacco use are major risk factors for most polygenic conditions that are more prevalent in males but, being modifiable, these factors are not necessarily the reason to exclude an embryo from being transferred.

## Ethical and social implications

### Autonomy

There is nothing inherently unethical in wanting one’s child to have the best possible life. Disease prevention, even decades into the future, would certainly be consistent with this desire. It has been argued that the principle of procreative beneficence (PPB) should guide these decisions:

Couples (or single reproducers) should select the child, of the possible children they could have, who is expected to have the best life, or at least as good a life as the others, based on the relevant, available information. (Savulescu, 2001)

There is a strong opposition to applying this principle to embryo selection (Herissone-Kelly, 2006; Bennett, 2009; Petersen, 2015) and various counterarguments have been proposed. Nevertheless, the PPB has an intuitive appeal, if not in academic circles then certainly to the broader public. Parents commonly go to extraordinary lengths to ensure the health, safety and future success of their children. It is equally clear that at least some prospective parents would embrace an opportunity to select the ‘best’ possible child. The widely accepted doctrine of procreative autonomy certainly supports prospective parents’ right to do so, not necessarily making it ethically obligatory. Thus, if information is available that may influence parental decision, it should be disclosed. Likewise, if there is a test available that may provide such information, it should also be discussed, and the prospective parents should be free to choose whether to take it up. The issue is not primarily whether a validated test be made available and the information derived provided; rather, the issue is that the information derived from PRS-ES is commonly presented in a subjective fashion that promotes its purported benefits and minimizes its multiple limitations, some of which are addressed above (Pagnaer *et al.*, 2021; Turley *et al.*, 2021). Biased information compromises autonomy by causing people to make decisions they would not endorse if they had all relevant information, ultimately resulting in choices that may not be in one’s best interests, nor in the best interests of one’s child (Blumenthal-Barby, 2016). An additional problem is how to accurately communicate all the uncertainties associated with this novel technology, considering that even the experts disagree on its utility (Kumar *et al.*, 2021; Treff *et al.*, 2021). Therefore, one might conclude that the application of PRS-ES is not suitable for implementation outside appropriately conducted research studies, where information provision and consent processes are more robust.

### Beneficence and non-maleficence (harms and benefits)

The considerations of beneficence and non-maleficence are relevant. Medical interventions have risks. The net intended benefit must outweigh those risks for an intervention to be ethically justified (Gillon, 1994). As demonstrated in the discussion above, in relation to clinical utility of PRS-ES the benefit of this intervention is uncertain at best. There are also recognized risks of its widespread implementation, which may make this technology unsuitable for clinical use. Risks to the prospective parents and to the future offspring must be distinguished. Possible negative impact of PRS-ES on a communal level will also be considered.

At present, PRS-ES is marketed to patients who undergo IVF and embryo biopsy for unrelated reasons. Women undergo fertility treatment/embryo biopsy and accept its risks, irrespective of whether PRS-ES is applied. In these women, PRS-ES does not present additional risks. Indeed, it does not represent a risk to the future child if PGT is being performed for other valid reasons, for example testing for a monogenic condition or structural rearrangement.

However, one can envisage a scenario where some would choose to undertake this treatment purely to utilize PRS-ES. The IVF process is associated with potentially serious risks (Blumenfeld, 2018; Farhud *et al.*, 2019; Lattová *et al.*, 2019), which would be accepted by potential parents in the hope of providing a meaningful benefit to their future children. Another possibility is that prospective parents would choose to subject their embryos to trophectoderm biopsy for the sole purpose of PRS-ES. The biopsy process may decrease their overall chances of pregnancy (Homer, 2019; Orvieto and Gleicher, 2020; Yan *et al.*, 2021) and may result in further need for IVF treatments, since it is likely that many good quality embryos will be required to make the choice between them meaningful. Furthermore, prospective parents may be faced with difficult decisions about the future health of their children. PRS-ES may be used to assess the risk of multiple conditions concurrently. All tested embryos will have a different risk profile and a choice will need to be made as to which embryo is to be transferred. How does one choose between an embryo that has a 5% chance of developing type 2 diabetes by the age of 50 years and an embryo that has a 10% chance of developing Alzheimer’s disease by the age of 80 years? Some providers utilize quality-adjusted life-year calculations to assess the overall theoretical disease burden for a particular embryo. It is highly questionable whether this population-based matrix of overall health and well-being is suitable for this indication and may in fact add an extra level of uncertainty, the extent of which is difficult to quantify. This could lead to ‘choice overload’ and decision-making paralysis, where people avoid making a choice entirely (Hadar and Sood, 2014). This is exactly the situation described in the first report of the PRS-ES, where all five tested embryos had an elevated risk of breast cancer and the prospective parents decided against transferring any of them (Treff *et al.*, 2019a). Furthermore, the choices that prospective parents will have to make will be based on personal experiences and the perceived disease severity at present. It is possible that diseases considered severe currently may be easily treatable in the future. The choice made today may not necessarily be the best one in the long run. Parents will have to live with the knowledge that they may have chosen the “wrong” embryo for the rest of their lives. This could potentially result in decision regret and long-term psychological morbidity as well as an altered child-parent relationship.

The harms to the offspring relate mostly to situations where either ART or embryo biopsy are undertaken solely for the purpose of PRS-ES. This is because should trophectoderm biopsy be required for unrelated reasons and PRS-ES is utilized as an ‘add-on’, the embryo and future person are not exposed to additional risks. However, if IVF and PRS-ES are undertaken solely for the purposes of PRS-ES, the future person could, in one sense, be harmed by PRS if that procedure exposes him or her to avoidable risks. For example, PGT has been associated with certain uncommon side effects in pregnancy (Makhijani *et al.*, 2021), which may have long-term consequences (Manna *et al.*, 2022). Multiple medical conditions also appear to be more prevalent in ART-conceived individuals compared to their naturally conceived peers (Chen and Heilbronn, 2017; von Wolff and Haaf, 2020; Luke, 2021). These include adverse perinatal outcomes (preterm birth, low birthweight) (Schieve *et al.*, 2004; Pinborg *et al.*, 2013), birth defects (minor and major malformations and imprinting disorders) (Hansen *et al.*, 2002; El-Chaar *et al.*, 2009), certain rare malignancies (Källén *et al.*, 2005; Williams *et al.*, 2013) as well as, possibly, the late onset polygenic conditions that PRS-ES is supposed to address (Ceelen *et al.*, 2007; *al.*, 2008; Pontesilli *et al.*, 2015). These risks are quite small, likely to be in the order of 1% (Luke *et al.*, 2020) and most patients would accept such risk if the alternative is not to have a genetic progeny. However, a future child may have an interest in PGT not being performed (Amor *et al.*, 2022) because it represents risks and provides no benefits. Therefore, to undertake fertility treatment for the sole purpose of selecting an embryo that has marginally lower risk of developing a late-onset polygenic condition appears counterproductive and is likely to result in an overall reduced chance of a healthy life. A similar argument can be made in relation to trophectoderm biopsy, which itself appears to produce higher risks of adverse outcomes in an offspring (Cimadomo *et al.*, 2016; Zacchini *et al.*, 2017; Tocci, 2020). Therefore, an offspring that is born as a result of PRS-ES, where ART is undertaken for the singular purpose of embryo selection based on this technology, may end up having a less healthy life, compared to a naturally conceived individual.

The issue of pleiotropy deserves special consideration. It can be defined as a tendency of a single genotype to affect multiple phenotypes (Watanabe *et al.*, 2019). It is not surprising that the risk of type 1 diabetes influences the risk of heart disease and stroke, as it is a recognized risk factor for both. There are more complex interactions that are well recognized, especially in the field of psychogenetics: schizophrenia and creativity (Acar *et al.*, 2018) or bipolar disorder and educational attainment (Turley *et al.*, 2021). These interactions are not well understood and therefore it is possible that selecting for or against one risk may inadvertently alter the risk of an entirely unexpected outcome. The actual outcomes will not be known for decades and therefore the balance of outcomes cannot be easily assessed. Pleiotropy introduces yet another level of uncertainty where the use of PRS-ES may be either beneficial, neutral or detrimental.

### Justice (social implications)

To consider the social implications of PRS-ES, one must assume that implementation of this technology will result in a meaningful decrease in the risk of developing polygenic conditions compared to embryo selection based on an alternative criterion. As we argued above, this cannot be easily demonstrated and considering the insurmountable uncertainties and demonstratable logical limitations, such as the impact of embryo sex on the PRS-ES results, overall benefit is uncertain at best. For the sake of argument, let’s assume that the benefits outweigh the risks on an individual level. There remain significant concerns in relation to possible effects of this technology on society. Unequal access may exacerbate pre-existing social inequalities. PRS-ES for intelligence, educational achievement, or income echoes eugenic policies of the past, where ‘undesirable’ traits were to be eliminated from the gene pool. Embryo selection based on physical features, such as skin or eye colour, height or facial features, may entrench racial stereotypes, further increasing prejudice and exacerbating discrimination (Lázaro-Muñoz *et al.*, 2021). Furthermore, it is possible that if this technology becomes widespread, it may result in significant demographic alterations (Bu *et al.*, 2014), thereby decreasing population diversity, making it less able to meet future challenges (Gyngell, 2012). Widespread adoption of this technology may also inadvertently alter the sex ratio at birth owing to recognized gender differences in the prevalence of most polygenic conditions. Human sex ratios have remained remarkably consistent through time (1 to 1), and there is concern that skewing the sex ratio would lead to unpredictable social consequences and have a negative effect on the birth rate (Hesketh and Xing, 2006).

## Conclusion

PRS are derived from complex statistical modelling, based on GWAS, using SNP arrays. They are in the early stages of development and clinical applications are currently limited. It has been suggested that PRS assessment can be applied for embryo selection, to allow individuals and couples to choose to have children with the theoretical least possible chance of developing certain late-onset conditions. The same technology can be used to select embryos based on non-disease characteristics, such as intelligence, skin colour or height, among others. There are well-defined criteria for the evaluation of genetic tests, which commonly involve assessment of analytic and clinical validity, clinical utility, and its social and ethical implications. Analytic validity of PRS-ES is currently unproven. Clinical validity cannot be demonstrated since the test’s outcomes will not manifest themselves for decades. Owing to limitations inherent in constructing PRS, it appears possible that changing environmental influences may render PRS-ES clinically invalid. Clinical utility is likewise highly questionable, and it is likely that the application of PRS-ES will not produce benefits that are meaningful. There are risks associated with the uptake of this technology, especially if ART and/or embryo biopsy is undertaken for the sole purpose of PRS-ES. This risk applies to prospective parents, their offspring and society in general. Numerous ethical concerns remain unanswered and further research and widespread consultations are urgently required. Owing to significant uncertainty and possible harms associated with its implementation, PRS-ES should not be offered outside of the research setting at present.

## Authors’ roles

All authors participated in the conception, critical revision for important intellectual content and final approval of the manuscript. The initial draft was written by A/Prof Alex Polyakov.

## Funding

No specific funding was received for this project.

## Conflict of interest

Prof. J.S. is a Partner Investigator on an Australian Research Council grant, which has partial funding from Illumina, although the funds are not paid to him or his institution but to a collaborator’s institution. He is a member of Bayer Bioethics Committee.

## References

[deac159-B1] Acar S , Chen X, Cayirdag N. Schizophrenia and creativity: a meta-analytic review. Schizophr Res 2018;195:23–31. 28867517 10.1016/j.schres.2017.08.036

[deac159-B2] Amor DJ , Savulescu J, Wilkins-Haug L. ISPD 2021 debate – All in vitro fertilization cycles should involve pre-implantation genetic testing to improve fetal health and pregnancy outcomes. Prenat Diagn 2022;doi:10.1002/pd.6156. 10.1002/pd.6156 35470429

[deac159-B3] Australian Institute of Health and Welfare. Deaths in Australia. Canberra: AIHW, 2021.

[deac159-B4] Bennett R. The fallacy of the principle of procreative beneficence. Bioethics 2009;23:265–273. 18477055 10.1111/j.1467-8519.2008.00655.x

[deac159-B5] Bloomberg News. Picking embryos with best health odds sparks new DNA debate, 2021, September 17. https://www.bloomberg.com/news/articles/2021-09-17/picking-embryos-with-best-health-odds-sparks-new-dna-debate (15 January 2022, date last accessed).

[deac159-B6] Blumenfeld Z. The ovarian hyperstimulation syndrome. Vitam Horm 2018;107:423–451. 29544639 10.1016/bs.vh.2018.01.018

[deac159-B7] Blumenthal-Barby JS. Biases and heuristics in decision making and their impact on autonomy. Am J Bioeth 2016;16:5–15. 27111357 10.1080/15265161.2016.1159750

[deac159-B8] Botkin JR. Assessing the new criteria for newborn screening. Health Matrix Clevel 2009;19:163–186. 19459543

[deac159-B9] Bu Z , Chen ZJ, Huang G, Zhang H, Wu Q, Ma Y, Shi J, Xu Y, Zhang S, Zhang C et al Live birth sex ratio after in vitro fertilization and embryo transfer in China—an analysis of 121,247 babies from 18 centers. PLoS One 2014;9:e113522. 25412419 10.1371/journal.pone.0113522 PMC4239103

[deac159-B10] Burke W , Atkins D, Gwinn M, Guttmacher A, Haddow J, Lau J, Palomaki G, Press N, Richards CS, Wideroff L et al Genetic test evaluation: information needs of clinicians, policy makers, and the public. Am J Epidemiol 2002;156:311–318. 12181100 10.1093/aje/kwf055

[deac159-B11] Ceelen M , van Weissenbruch MM, Roos JC, Vermeiden JP, van Leeuwen FE, Delemarre-van de Waal HA. Body composition in children and adolescents born after in vitro fertilization or spontaneous conception. J Clin Endocrinol Metab 2007;92:3417–3423. 17595253 10.1210/jc.2006-2896

[deac159-B12] Ceelen M , van Weissenbruch MM, Vermeiden JP, van Leeuwen FE, Delemarre-van de Waal HA. Cardiometabolic differences in children born after in vitro fertilization: follow-up study. J Clin Endocrinol Metab 2008;93:1682–1688. 18285409 10.1210/jc.2007-2432

[deac159-B13] Chambers GM , Paul RC, Harris K, Fitzgerald O, Boothroyd CV, Rombauts L, Chapman MG, Jorm L. Assisted reproductive technology in Australia and New Zealand: cumulative live birth rates as measures of success. Med J Aust 2017;207:114–118. 28764619 10.5694/mja16.01435

[deac159-B14] Chen M , Heilbronn LK. The health outcomes of human offspring conceived by assisted reproductive technologies (ART). J Dev Orig Health Dis 2017;8:388–402. 28416029 10.1017/S2040174417000228

[deac159-B15] Cimadomo D , Capalbo A, Ubaldi FM, Scarica C, Palagiano A, Canipari R, Rienzi L. The impact of biopsy on human embryo developmental potential during preimplantation genetic diagnosis. Biomed Res Int 2016;2016:7193075. 26942198 10.1155/2016/7193075 PMC4749789

[deac159-B16] Delaneau O , Zagury JF, Robinson MR, Marchini JL, Dermitzakis ET. Accurate, scalable and integrative haplotype estimation. Nat Commun, 2019;10:5436. 31780650 10.1038/s41467-019-13225-y PMC6882857

[deac159-B17] El-Chaar D , Yang Q, Gao J, Bottomley J, Leader A, Wen SW, Walker M. Risk of birth defects increased in pregnancies conceived by assisted human reproduction. Fertil Steril 2009;92:1557–1561. 18973885 10.1016/j.fertnstert.2008.08.080

[deac159-B18] Farhud DD , Zokaei S, Keykhaei M, Yeganeh MZ. Strong evidences of the ovarian carcinoma risk in women after IVF treatment: a review article. Iran J Public Health 2019;48:2124. PMC6974869 31993380

[deac159-B19] Genomic Prediction Clinical Laboratory. LifeView PGT-P preimplantation genetic testing for polygenic disorders, 2022, January 15. https://anyflip.com/rough/uvwc (19 January 2022, date last accessed).

[deac159-B20] Gillon R. Medical ethics: four principles plus attention to scope. BMJ 1994;309:184–188. 8044100 10.1136/bmj.309.6948.184 PMC2540719

[deac159-B21] Golan D , Lander ES, Rosset S. Measuring missing heritability: Inferring the contribution of common variants. Proc Natl Acad Sci USA 2014;111:E5272–E5281. 25422463 10.1073/pnas.1419064111 PMC4267399

[deac159-B22] Gyngell C. Enhancing the species: genetic engineering technologies and human persistence. Philos Technol 2012;25:495–512.

[deac159-B23] Hadar L , Sood S. When knowledge is demotivating: subjective knowledge and choice overload. Psychol Sci 2014;25:1739–1747. 25037963 10.1177/0956797614539165

[deac159-B24] Hansen M , Kurinczuk JJ, Bower C, Webb S. The risk of major birth defects after intracytoplasmic sperm injection and in vitro fertilization. N Engl J Med 2002;346:725–730. 11882727 10.1056/NEJMoa010035

[deac159-B25] Herissone-Kelly P. Procreative beneficence and the prospective parent. J Med Ethics 2006;32:166–169. 16507665 10.1136/jme.2005.012369 PMC2564476

[deac159-B26] Hesketh T , Xing ZW. Abnormal sex ratios in human populations: Causes and consequences. Proc Natl Acad Sci USA 2006;103:13271–13275. 16938885 10.1073/pnas.0602203103 PMC1569153

[deac159-B27] Holtzman NA , Watson MS. Promoting Safe and Effective Genetic Testing in the United States: final Report of the Task Force on Genetic Testing. Baltimore: Johns Hopkins University Press, 1998. 10795196

[deac159-B28] Homer HA. Preimplantation genetic testing for aneuploidy (PGT-A): the biology, the technology and the clinical outcomes. Aust N Z J Obst Gynaecol 2019;59:317–324. 30811595 10.1111/ajo.12960

[deac159-B29] Källén B , Finnström O, Nygren KG, Olausson PO. In vitro fertilization in Sweden: child morbidity including cancer risk. Fertil Steril 2005;84:605–610. 16169392 10.1016/j.fertnstert.2005.03.035

[deac159-B30] Karavani E , Zuk O, Zeevi D, Barzilai N, Stefanis NC, Hatzimanolis A, Smyrnis N, Avramopoulos D, Kruglyak L, Atzmon G et al Screening human embryos for polygenic traits has limited utility. Cell 2019;179:1424–1435.e8. 31761530 10.1016/j.cell.2019.10.033 PMC6957074

[deac159-B31] Kendler KS , Aggen SH, Gillespie N, Krueger RF, Czajkowski N, Ystrom E, Reichborn-Kjennerud T. The structure of genetic and environmental influences on normative personality, abnormal personality traits, and personality disorder symptoms. Psychol Med 2019;49:1392–1399. 30722797 10.1017/S0033291719000047 PMC6520133

[deac159-B32] Khoury MJ , Janssens AC, Ransohoff DF. How can polygenic inheritance be used in population screening for common diseases? Genet Med 2013;15:437–443. 23412608 10.1038/gim.2012.182 PMC4692802

[deac159-B33] King JR , Notarangelo LD, Hammarström L. An appraisal of the Wilson & Jungner criteria in the context of genomic-based newborn screening for inborn errors of immunity. J Allergy Clin Immunol 2021;147:428–438. 33551024 10.1016/j.jaci.2020.12.633 PMC8344044

[deac159-B34] Kling D , Phillips C, Kennett D, Tillmar A. Investigative genetic genealogy: current methods, knowledge and practice. Forensic Sci Int Genet 2021;52:102474. 33592389 10.1016/j.fsigen.2021.102474

[deac159-B35] Konuma T , Okada Y. Statistical genetics and polygenic risk score for precision medicine. Inflamm Regen 2021;41:18. 34140035 10.1186/s41232-021-00172-9 PMC8212479

[deac159-B36] Kumar A , Im K, Banjevic M, Ng PC, Tunstall T, Garcia G, Galhardo L, Sun J, Schaedel ON, Levy B et al Whole-genome risk prediction of common diseases in human preimplantation embryos. Nat Med 2022;28:513–516. 35314819 10.1038/s41591-022-01735-0 PMC8938270

[deac159-B37] Kumar A , Im K, Rabinowitz M. Use of polygenic scores to select embryos. N Engl J Med 2021;385:1726–1727. 34706186 10.1056/NEJMc2113013

[deac159-B38] Lattová V , Dostál J, Vodička J, Procházka M. The risk of thromboembolism in relation to in vitro fertilization. Ceska Gynekol 2019;84:229–232. 31324115

[deac159-B39] Lázaro-Muñoz G , Pereira S, Carmi S, Lencz T. Screening embryos for polygenic conditions and traits: ethical considerations for an emerging technology. Genet Med 2021;23:432–434. 33106616 10.1038/s41436-020-01019-3 PMC7936952

[deac159-B40] Lencz T , Backenroth D, Granot-Hershkovitz E, Green A, Gettler K, Cho JH, Weissbrod O, Zuk O, Carmi S. Utility of polygenic embryo screening for disease depends on the selection strategy. Elife 2021;10:e64716. 34635206 10.7554/eLife.64716 PMC8510582

[deac159-B41] Lewis CM , Vassos E. Polygenic risk scores: from research tools to clinical instruments. Genome Med 2020;12:44. 32423490 10.1186/s13073-020-00742-5 PMC7236300

[deac159-B42] Luke B. The health of in vitro fertilization-conceived children: the blind men and the elephant. Fertil Steril 2021;116:1524–1525. 34743911 10.1016/j.fertnstert.2021.09.038 PMC9761789

[deac159-B43] Luke B , Brown MB, Nichols HB, Schymura MJ, Browne ML, Fisher SC, Forestieri NE, Rao C, Yazdy MM, Gershman ST et al Assessment of birth defects and cancer risk in children conceived via in vitro fertilization in the US. JAMA Netw Open 2020;3:e2022927. 33119107 10.1001/jamanetworkopen.2020.22927 PMC7596575

[deac159-B44] Makhijani R , Bartels CB, Godiwala P, Bartolucci A, DiLuigi A, Nulsen J, Grow D, Benadiva C, Engmann L. Impact of trophectoderm biopsy on obstetric and perinatal outcomes following frozen-thawed embryo transfer cycles. Hum Reprod 2021;36:340–348. 33313768 10.1093/humrep/deaa316

[deac159-B45] Manna C , Lacconi V, Rizzo G, Lorenzo AD, Massimiani M. Placental dysfunction in assisted reproductive pregnancies: perinatal, neonatal and adult life outcomes. Int J Mol Sci 2022;23:659. 35054845 10.3390/ijms23020659 PMC8775397

[deac159-B46] Manolio TA , Collins FS, Cox NJ, Goldstein DB, Hindorff LA, Hunter DJ, McCarthy MI, Ramos EM, Cardon LR, Chakravarti A et al Finding the missing heritability of complex diseases. Nature 2009;461:747–753. 19812666 10.1038/nature08494 PMC2831613

[deac159-B47] Mokdad AH , Ballestros K, Echko M, Glenn S, Olsen HE, Mullany E, Lee A, Khan AR, Ahmadi A, Ferrari AJ et al; US Burden of Disease Collaborators. The state of US Health, 1990–2016: burden of diseases, injuries, and risk factors among US states. JAMA 2018;319:1444–1472. 29634829 10.1001/jama.2018.0158 PMC5933332

[deac159-B48] Munné S , Kaplan B, Frattarelli JL, Child T, Nakhuda G, Shamma FN, Silverberg K, Kalista T, Handyside AH, Katz-Jaffe M et al; STAR Study Group. Preimplantation genetic testing for aneuploidy versus morphology as selection criteria for single frozen-thawed embryo transfer in good-prognosis patients: a multicenter randomized clinical trial. Fertil Steril 2019;112:1071–1079.e77. 31551155 10.1016/j.fertnstert.2019.07.1346

[deac159-B49] Orchid Health. Identify Your Healthiest Embryo. 2022, February 12. https://www.orchidhealth.com/embryo (15 January 2022, date last accessed).

[deac159-B50] Orfila F , Ferrer M, Lamarca R, Tebe C, Domingo-Salvany A, Alonso J. Gender differences in health-related quality of life among the elderly: the role of objective functional capacity and chronic conditions. Soc Sci Med 2006;63:2367–2380. 16884840 10.1016/j.socscimed.2006.06.017

[deac159-B51] Orvieto R , Gleicher N. Preimplantation genetic testing for aneuploidy (PGT-A)-finally revealed. J Assist Reprod Genet 2020;37:669–672. 32008181 10.1007/s10815-020-01705-w PMC7125263

[deac159-B52] Pagliardini L , Viganò P, Alteri A, Corti L, Somigliana E, Papaleo E. Shooting STAR: reinterpreting the data from the ‘Single Embryo TrAnsfeR of Euploid Embryo’ randomized clinical trial. Reprod Biomed Online 2020;40:475–478. 32273162 10.1016/j.rbmo.2020.01.015

[deac159-B53] Pagnaer T , Siermann M, Borry P, Tšuiko O. Polygenic risk scoring of human embryos: a qualitative study of media coverage. BMC Med Ethics 2021;22:125. 34537037 10.1186/s12910-021-00694-4 PMC8449454

[deac159-B54] Peters BA , Kermani BG, Alferov O, Agarwal MR, McElwain MA, Gulbahce N, Hayden DM, Tang YT, Zhang RY, Tearle R et al Detection and phasing of single base de novo mutations in biopsies from human in vitro fertilized embryos by advanced whole-genome sequencing. Genome Res 2015;25:426–434. 25672852 10.1101/gr.181255.114 PMC4352880

[deac159-B55] Petersen TS. On the partiality of procreative beneficence: a critical note. J Med Ethics 2015;41:771–774. 25907895 10.1136/medethics-2014-102420

[deac159-B56] Petros M. Revisiting the Wilson-Jungner criteria: how can supplemental criteria guide public health in the era of genetic screening? Genet Med 2012;14:129–134. 22237442 10.1038/gim.0b013e31823331d0

[deac159-B57] Pinborg A , Wennerholm UB, Romundstad LB, Loft A, Aittomaki K, Söderström-Anttila V, Nygren KG, Hazekamp J, Bergh C. Why do singletons conceived after assisted reproduction technology have adverse perinatal outcome? Systematic review and meta-analysis. Hum Reprod Update 2013;19:87–104. 23154145 10.1093/humupd/dms044

[deac159-B58] Pontesilli M , Painter RC, Grooten IJ, van der Post JA, Mol BW, Vrijkotte TG, Repping S, Roseboom TJ. Subfertility and assisted reproduction techniques are associated with poorer cardiometabolic profiles in childhood. Reprod Biomed Online 2015;30:258–267. 25592973 10.1016/j.rbmo.2014.11.006

[deac159-B59] Savulescu J. Procreative beneficence: why we should select the best children. Bioethics 2001;15:413–426. 12058767 10.1111/1467-8519.00251

[deac159-B60] Schieve LA , Rasmussen SA, Buck GM, Schendel DE, Reynolds MA, Wright VC. Are children born after assisted reproductive technology at increased risk for adverse health outcomes? Obstet Gynecol 2004;103:1154–1163. 15172847 10.1097/01.AOG.0000124571.04890.67

[deac159-B61] Steptoe PC , Edwards RG. Birth after the reimplantation of a human embryo. Lancet 1978;2:366. 10.1016/s0140-6736(78)92957-4 79723

[deac159-B62] Tada H , Fujino N, Hayashi K, Kawashiri MA, Takamura M. Human genetics and its impact on cardiovascular disease. J Cardiol 2021;79:233–239. 34551866 10.1016/j.jjcc.2021.09.005

[deac159-B63] Teutsch SM , Bradley LA, Palomaki GE, Haddow JE, Piper M, Calonge N, Dotson WD, Douglas MP, Berg AO; EGAPP Working Group. The evaluation of genomic applications in practice and prevention (EGAPP) initiative: methods of the EGAPP Working Group. Genet Med 2009;11:3–14. 18813139 10.1097/GIM.0b013e318184137c PMC2743609

[deac159-B64] Tocci A. The unknown human trophectoderm: implication for biopsy at the blastocyst stage. J Assist Reprod Genet 2020;37:2699–2711. 32892265 10.1007/s10815-020-01925-0 PMC7642161

[deac159-B65] Treff NR , Eccles J, Lello L, Bechor E, Hsu J, Plunkett K, Zimmerman R, Rana B, Samoilenko A, Hsu S et al Utility and first clinical application of screening embryos for polygenic disease risk reduction. Front Endocrinol (Lausanne) 2019a;10:845. 31920964 10.3389/fendo.2019.00845 PMC6915076

[deac159-B66] Treff NR , Tellier L, Hsu SDH. Use of polygenic scores to select embryos. N Engl J Med 2021;385:1727. 10.1056/NEJMc2113013 34706187

[deac159-B67] Treff NR , Zimmerman R, Bechor E, Hsu J, Rana B, Jensen J, Li J, Samoilenko A, Mowrey W, Van Alstine J et al Validation of concurrent preimplantation genetic testing for polygenic and monogenic disorders, structural rearrangements, and whole and segmental chromosome aneuploidy with a single universal platform. Eur J Med Genet2019b;62:103647. 31026593 10.1016/j.ejmg.2019.04.004

[deac159-B68] Turley P , Meyer MN, Wang N, Cesarini D, Hammonds E, Martin AR, Neale BM, Rehm HL, Wilkins-Haug L, Benjamin DJ et al Problems with using polygenic scores to select embryos. N Engl J Med 2021;385:78–86. 34192436 10.1056/NEJMsr2105065 PMC8387884

[deac159-B69] von Wolff M , Haaf T. In vitro fertilization technology and child health. Dtsch Arztebl Int 2020;117:23–30. 32031509 10.3238/arztebl.2020.0023 PMC7026576

[deac159-B70] Wald NJ , Old R. The illusion of polygenic disease risk prediction. Genet Med 2019;21:1705–1707. 30635622 10.1038/s41436-018-0418-5

[deac159-B71] Wallis JM. Is it ever morally permissible to select for deafness in one's child? Med Health Care Philos 2020;23:3–15. 31542873 10.1007/s11019-019-09922-6 PMC7040060

[deac159-B72] Watanabe K , Stringer S, Frei O, Umićević Mirkov M, de Leeuw C, Polderman TJC, van der Sluis S, Andreassen OA, Neale BM, Posthuma D. A global overview of pleiotropy and genetic architecture in complex traits. Nat Genet 2019;51:1339–1348. 31427789 10.1038/s41588-019-0481-0

[deac159-B73] Williams CL , Bunch KJ, Stiller CA, Murphy MF, Botting BJ, Wallace WH, Davies M, Sutcliffe AG. Cancer risk among children born after assisted conception. N Engl J Med 2013;369:1819–1827. 24195549 10.1056/NEJMoa1301675

[deac159-B74] Wilson JMG , Jungner G. Principles and Practice of Screening for Disease. Geneva: World Health Organization, 1968.

[deac159-B75] Yan J , Qin Y, Zhao H, Sun Y, Gong F, Li R, Sun X, Ling X, Li H, Hao C et al Live birth with or without preimplantation genetic testing for aneuploidy. N Engl J Med 2021;385:2047–2058. 34818479 10.1056/NEJMoa2103613

[deac159-B76] Zacchini F , Arena R, Abramik A, Ptak GE. Embryo biopsy and development: the known and the unknown. Reproduction 2017;154:R143–R148. 28851825 10.1530/REP-17-0431

[deac159-B77] Zong C , Lu S, Chapman AR, Xie XS. Genome-wide detection of single-nucleotide and copy-number variations of a single human cell. Science 2012;338:1622–1626. 23258894 10.1126/science.1229164 PMC3600412

